# Retrospective Assessment of Antibiotics Prescribing at Public Primary Healthcare Facilities in Addis Ababa, Ethiopia

**DOI:** 10.1155/2018/4323769

**Published:** 2018-02-28

**Authors:** Fikru Worku, Dagmawit Tewahido

**Affiliations:** Addis Continental Institute of Public Health, P.O. Box 26751/1000, Addis Ababa, Ethiopia

## Abstract

*Background. *Antibiotic overprescribing is the major driving force for the emergence of antibiotics resistance. The aim of this study was to assess antibiotics prescribing at primary healthcare facilities in Addis Ababa, Ethiopia.* Methods. *The study was conducted in six public health centers found in Addis Ababa City. Data was collected retrospectively from a total of 900 prescriptions and selected medical charts of patients in the health centers in 2016. Data was entered and analyzed using EPI Info 7 and SPSS 20, respectively. Descriptive statistics and logistic regression analysis were used to analyze the data.* Results. *One or more antibiotics were prescribed in 56.0% of the prescriptions. Antibiotics accounted for 46.0% of the total cost of medicines prescribed. Amoxicillin was the most frequently (44.8%) prescribed antibiotic and upper respiratory tract infection was the most common (24.5%) diagnosis for prescribing antibiotics. Laboratory investigation was done for only about 27% of the cases for which antibiotics were prescribed.* Conclusion. *There was a high rate of antibiotics prescribing in the health centers often empirically which might exacerbate the antimicrobial resistance situation in the country. Large-scale study should be conducted to fully understand the prescribing pattern and identify the associated factors thereby design and implement appropriate interventions.

## 1. Introduction

The success of antimicrobials against disease-causing microbes is among modern medicine's greatest achievements. After more than 70 years of widespread use, however, many antimicrobials are not as effective as they used to be due to the emergence and spread of antimicrobial resistance (AMR) [1]. AMR, especially resistance to antibacterials, has become a worldwide challenge to public health resulting in treatment failure and increased mortality and costs of care. Deaths from drug-resistant infections are projected to increase from the current 700,000 to 10 million annually, and cost estimates are expected to be as high as US$ 100 trillion worldwide by 2050. The prospect of the world entering a “postantibiotic era,” where common infections can no longer be cured, is therefore a real possibility [2].

There is wide consensus that the main factor driving AMR development is the overprescribing of antibiotics [3]. The more the antibiotics are used, particularly when misused, the greater the selective pressure placed on bacteria to acquire resistance genes, hence the need to limit the use of these medicines to what is necessary and appropriate [4]. Studies conducted in Ethiopia have revealed that most bacteria that cause infections to human beings and animals have developed considerable degree of resistance to commonly used first-line antibiotics [5, 6]. The most effective strategy for combating antibiotic resistance is rational antibiotic prescribing [7]. Antibiotics are those antimicrobial agents used for the prevention and treatment of infections caused by bacteria and AMR refers to resistance to antibiotics (antibacterials).

The percentage use of antibiotics has been used as a key indicator to determine whether the use of antibiotics is appropriate or not. The World Health Organization (WHO) recommends percentage of encounters with one or more antibiotics prescribed to be less than 30% for general outpatients in primary healthcare facilities [8]. National [9, 10] as well as facility-specific general prescribing studies conducted in Ethiopia have indicated high rate of antibiotics prescribing. The percentage of antibiotics prescribed was 58 and 60% in the national pharmaceutical sector assessments conducted in 2003 [9] and 2010 [10], respectively. Very high rate (82.5%) of antibiotics prescribing was reported from studies conducted in health centers found in Somali Regional State, Eastern Ethiopia [11]. A relatively higher percentage (63.0%) of antibiotics prescribing was also reported by a study conducted in primary healthcare facilities in Wolkite Town, Southwest Ethiopia [12]. Lower percentage (41.3%) of antibiotic prescribing was reported from public health centers found in Bahir Dar City [13].

The Ethiopian healthcare system is structured in a three-tier system: primary, secondary, and tertiary level of care. The primary level of care includes primary hospital, health center, and health post [14]. This study was conducted at government-owned (public) health centers each of which provides both preventive and curative health services to an average population of 25,000. Health centers also serve as a referral center and practical attachment sites for Health Posts which are staffed with Health Extension Workers (HEWs) in rural areas.

Despite the alarming findings from general medicine use studies on the overuse of antibiotics, studies that focused on the rate and patterns of antibiotics prescribing at primary healthcare facilities have not been conducted so far in the country in general and in Addis Ababa in particular, to the knowledge of the authors. This study was, therefore, conducted to assess the rate and patterns of antibiotics prescribing at public health centers in Addis Ababa, Ethiopia. Findings of this study will serve as a basis for undertaking detailed criteria-based medicine use studies to check appropriateness of the commonly used antibiotics, identify the associated factors, and thereby optimize their use. This will contribute its part to the national effort on AMR Prevention and Containment.

## 2. Methods and Materials

### 2.1. Study Setting

The study was conducted at selected public health centers found in Addis Ababa City Administration. Addis Ababa is the capital city of Ethiopia. The city has 10 subcities with a total population of 3,352,000 (1,587,000 males and 1,765,000 females) according to the population projection for 2016 of the 2007 National Population and Housing Census [15]. According to the information obtained from the City Administration Health Bureau, there are a total of 92 functional public health centers in the city. Twenty-six of these health centers have been providing service for many years before the new generation of health centers came into existence [16]. These first generation of health centers were those that have been providing service for at least 8 years prior to the study.

### 2.2. Study Design

This study was conducted through retrospective review of prescriptions and medical charts of patients.

### 2.3. Study Population

The study population constituted patients who got service at outpatient level in the selected public health centers in Addis Ababa during the year 2016 (January 1–December 31, 2016). Patients for whom one or more medicines were prescribed during working and emergency hours of working days were included in the study.

### 2.4. Sample Size and Sampling Procedures

The study was conducted in six of the 26 health centers (23.1%) that have long experience of service provision in the city. These health centers were selected from 6 of the 10 subcities found in the city (one health center per selected subcity). WHO recommends reviewing 100 prescriptions per health facility to describe or compare drug use by individual facilities and at least 600 prescriptions to describe the overall prescribing practice in a group of facilities [17]. In this study, a total of 150 prescriptions filled in 2016 were reviewed from each selected health center (total of 900 prescriptions). This sample size made it possible to make comparison among the health centers included in the study for some of the indicators.

This study required multistage sampling at 3 levels. In the first stage, six subcities (Addis Ketema, Arada, Bole, Gulele, Kolfe Keraniyo, and Kirkos) were selected by simple random sampling method (lottery method). In the second stage, one health center (HC) was selected by simple random sampling method (lottery method) from the list of long-experience health centers found in each selected subcity as per the data obtained from the Health Bureau. In the third stage, the working days during the year 2016 (January 1–December 31, 2016) were identified by date from a calendar and recorded on a sheet of paper. From the list of working days identified in the year, 150 working days were selected by systematic random sampling method. WHO recommends using 12-month prescribing data as a sampling frame so as to accommodate seasonal variations in disease and prescribing pattern [17].

Prescriptions dispensed during each day are collected according to their order of coming, packed and labeled on daily basis by the dispensers. Packs of prescriptions dispensed during each selected working date were retrieved from the dispensary and one prescription was taken by simple random sampling method (Lottery method) from each sampled date's prescription pack. While picking prescriptions from subsequent dates, effort was made to spread the prescriptions selected over different times of the day since prescriptions are dispensed and packed in the order they arrive in the dispensary; for the first selected date, prescription was picked from the beginning of the pack, for the second selected date from the middle, and so on as recommended by WHO [17]. No sampling was done for patient medical charts. Rather, medical charts of all patients included in the sample for whom one or more antibiotics were prescribed were considered for the study.

In the case of Selam Health Center where the recording system is electronic, the list of patients (by their registration number) who got service during all sampled date was retrieved from the system after getting access to the system. Then, one patient was selected by lottery method from the list of patients in all sampled working date.

### 2.5. Data Collection Tools and Procedures

Data was collected from prescriptions and patient medical charts using data abstraction form partly adapted from WHO prescribing indicators data collection form [17]. The form was developed so as to capture information on patient (age, sex, and registration number), date of prescribing, medicines and antibiotics prescribed, cost of medicines and antibiotics prescribed, route of administration, type and category of antibiotics prescribed, diagnosis, and laboratory investigations done. The necessary data was collected by three experienced clinically oriented pharmacists (one pharmacist for 2 health centers) each assisted by one pharmacist assigned from each health center. Data collectors were trained by the principal investigator on objectives of the study, the sampling procedure, the data abstraction form, and how to collect data from prescriptions and patient medical charts using the form.

The data abstraction form was pretested in two health centers prior to the actual study using 10 prescriptions and 5 patient medical charts in each health center and the necessary modifications were made on the form. The modifications made include the inclusion of diagnosis among the data to be collected from prescriptions as this was found possible and leaving the collection of information about the existing prescribers so as to retrospectively link with their prescribing practice as this was not found feasible. The two health centers were excluded from the study. The data was collected during January 2–20, 2017 under close supervision of the principal investigator.

From each sampled prescription, data on registration number, patient's age, sex, date of prescribing, number of medicines and antibiotics prescribed, costs of medicines, and cost of antibiotics were collected. From prescription containing one or more antibiotics, information on name of the antibiotic prescribed, routes of administration, and the diagnosis for which the antibiotic was prescribed (whenever available) was collected. Information on the diagnosis for which the antibiotics were prescribed (if not written on prescriptions), whether or not laboratory investigations were done, was collected from patient medical charts that were retrieved based on the registration numbers written on prescriptions.

Antibacterials belonging to the following categories of medicines in the List of Medicines for Health Centers in Ethiopia (3rd edition) were considered as antibiotics:* penicillins, other antibacterials, ophthalmic antibacterials, and topical antibacterials *[18].

### 2.6. Data Analysis

The data collected was entered into the data entry questionnaire developed on EPI Info version 7. It was then imported to SPSS version 20. All categorical data was labeled based on the label used in the EPI Info. One or more pieces of data that were not found from prescriptions or medical charts were considered as “missing” and labeled as such in the SPSS. Entries that were not applicable for one or more of the variables were also labeled as “missing.” Data cleaning was done in the EPI Info as well as after importing to SPSS by checking correctness of each of the entries made. Necessary corrective measures were taken when illogical entries were encountered. Descriptive statistical analysis was used to analyze most of the data at 95% CI.

The following indicators were determined from the data collected:

- *Average number of medicines per prescription: *calculated by dividing the total number of medicines prescribed by the number of prescriptions reviewed.
- *Average number of antibiotics per prescription*: calculated by dividing the total number of antibiotics prescribed by the total number of prescriptions reviewed.
- *Percentage of prescriptions with antibiotics prescribed: *calculated by dividing the number of prescriptions containing one or more antibiotics by the total number of prescriptions reviewed times 100.
- *Percentage of antibiotics prescribed: *calculated by dividing the total number of antibiotics prescribed by the total number of medicines prescribed times 100.
- *Average cost of medicines per prescription: *calculated by dividing the total cost of medicines prescribed by the number of prescriptions reviewed times 100.
- *Percentage cost of antibiotics: *calculated by dividing the total cost of antibiotics prescribed by the total cost of all medicines prescribed times 100.
- *Percentage of encounters with antibiotics prescribed after laboratory test*: calculated by dividing the number of encounters with laboratory investigation done by the total number of encounters with antibiotics prescribed times 100.
- *Percentage of antibiotics prescribed based on antimicrobial sensitivity test (AST) results*: calculated by dividing the number of antibiotics prescribed based on positive AST by the total number of antibiotics prescribed times 100.

Bivariate logistic regression analysis was conducted to check the association of patient-related and seasonal factors with antibiotics prescribing at 95% CI. The commonly prescribed category and type of antibiotics, routes of administration, and the common diagnosis for which antibiotics were prescribed were also determined.

### 2.7. Ethical Considerations

The research proposal was approved by the Ethical Review Board of Addis Continental Institute of Public Health. Ethical clearance was obtained from Ethical Clearance Committee of Addis Ababa City Administration Health Bureau and verbal consent was obtained from the management of each health center prior to data collection. To ensure confidentiality of patient-specific information, patient medical charts as well as prescriptions were handled with great care and all the data was collected within each health center's compound with the assistance of the assigned pharmacist from each health center. Patient-specific information like name were not recorded and not used in analysis.

## 3. Results

A total of 900 prescriptions were reviewed from the six health centers. Majority (61.0%) of the prescriptions were prescribed for females and for patients in the age group of 15–34 years (41.7%). With regard to season of prescribing, more or less similar numbers of prescriptions were taken from each quarter (Table 1).

### 3.1. Rate of Antibiotics Prescribing

**Figure 1:**
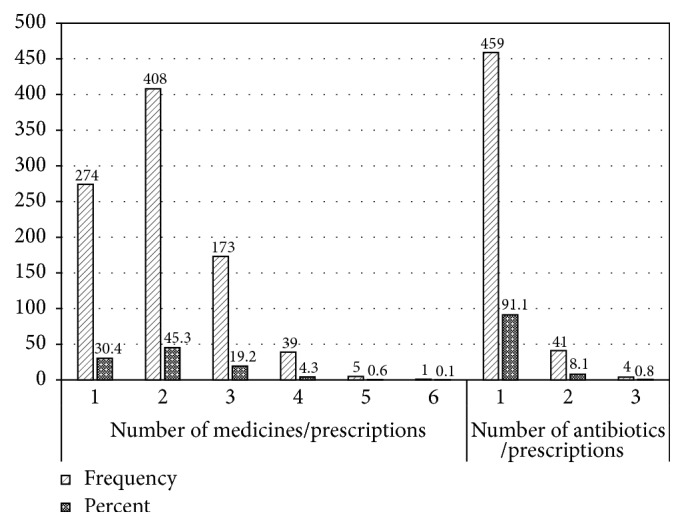
Number of medicines and antibiotics prescribed per prescription at health centers in Addis Ababa, January 1–December 31, 2016.

A total of 1796 medicines were prescribed in the 900 prescriptions with an average number of medicines per prescription of 2.0 (95% CI: 1.94, 2.05). This value ranged from 1.9 to 2.2 among the health centers. Majority of the prescriptions (45.3%) contained two medicines and about a quarter (24.3%) of the prescriptions contained 3 or more medicines. Five-hundred and four (56.0%) of the prescriptions contained one or more antibiotics (95% CI: 52.8, 59.2). This value ranged from 46.7% (Kirkos HC) to 67.3% (Addis Ketema HC) among the health centers surveyed. The average number of antibiotics per prescription was 0.65 (95% CI: 0.58, 0.65). The percentage of antibiotics per number of medicines prescribed was 30.8% (Figure 1).

Table 2 shows the rate of antibiotics prescribing by patient-related factors and season of prescribing. A relatively higher percentage of antibiotics were shown to be prescribed for female patients as compared to males in all age groups except in the age group of 55 years and above. Antibiotics prescribing rate showed decreasing pattern with increase in patient's age. A relatively lower percentage of antibiotics (50.7%) was prescribed during 2nd quarter of the year.

### 3.2. Antibiotics Prescribing Pattern

Table 3 shows the most frequently prescribed category and type of antibiotics. By antibiotics category, penicillins were the most frequently prescribed (51.9%) category of antibiotics followed by fluoroquinolones (18.3%) and sulphonamides (11.2%). These three antibiotic categories accounted for over 80% of the antibiotics prescribed. Amoxicillin was the most frequently prescribed antibiotics (44.8%) followed by ciprofloxacin (13.6%) and cotrimoxazole (11.2%).

Most of the antibiotics prescribed were for oral administration (94.8%) followed by topical (2.7%) and parenteral (2.5%) routes. Tetracycline, chloramphenicol, and gentamycin were the antibiotics prescribed for topical application (skin, eye, or ear infections) whereas ceftriaxone was the only antibiotic prescribed for parenteral administration.

**Figure 2:**
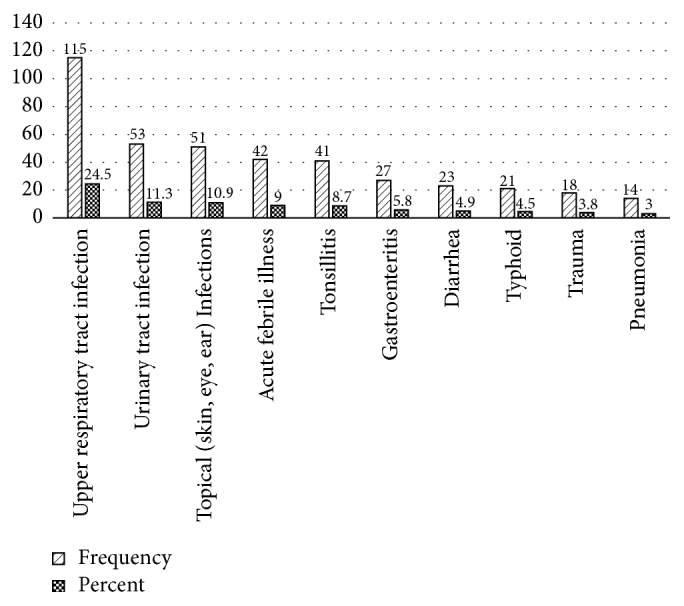
Top 10 disease conditions for which antibiotics were prescribed at health centers in Addis Ababa, January 1–December 31, 2016.

Of the 504 patients for whom one or more antibiotics were prescribed, it was possible to retrieve medical charts for 484 (96.0%) of them. Laboratory investigation was ordered for 134 (27.7%) of these patients (95% CI: 23.6–31.8) and AST was not done for any of the patients for whom antibiotics were prescribed. Information about the diagnosis for prescribing antibiotics was found for 466 (92.5%) of the patients for whom one or more antibiotics were prescribed. Upper respiratory tract infection (URTI) was the most frequent diagnosis (24.5%) followed by urinary tract infections (UTIs) (11.3%) and topical (skin, eye, and ear) infections (10.9%). Only 10 disease conditions accounted for 86.4% of the cases for antibiotics prescribing (Figure 2).

Table 4 shows the common diagnosis for frequently prescribed antibiotics as a single medicine. URTI (49.5%), pneumonia (28.6%), AFI (41.2%), AFI (80.8%), and diarrhea (29.4%) were the most common diagnosis for the prescribing of amoxicillin, amoxicillin + clavulanic acid, ciprofloxacin, doxycycline, and cotrimoxazole, respectively.

### 3.3. Cost of Antibiotics

Antibiotics accounted for 46.0% of the cost of medicines prescribed. The highest percentage of cost of antibiotics (22.7%) was taken by URTIs followed by topical infections (11.3%) and tonsillitis (8.6%). Only 5 types of diagnosis took about 59% of the cost of antibiotics prescribed: URTI (22.7%), topical infections (11.3%), tonsillitis (8.6%), UTI (8.2%), and AFI (7.8%). Prescribed alone, amoxicillin, amoxicillin + clavulanic acid, cotrimoxazole, ciprofloxacin, and cloxacillin constituted 43.5, 10.3, 7.4, 6.9, and 6.6% of the cost of antibiotics prescribed, respectively. Overall, these five antibiotics constituted 65.4% of the cost of antibiotics prescribed.

### 3.4. Factors Associated with Antibiotics Prescribing

Patient's age was found to have statistically significant association with antibiotics prescribing. The odds of prescribing antibiotics to patients in the age group of 0–14 years and 15–34 years were shown to be about 4 times and 2.5 times higher, respectively, than patients in the age group of 55 years and above. Patient's sex and prescribing quarter were not found to have statistically significant association with antibiotics prescribing (Table 5).

## 4. Discussion

This study aimed at assessing antibiotics prescribing (the rate and patterns of prescribing) at public (government-owned) primary healthcare facilities. These facilities provide primary healthcare service to the population of Addis Ababa and its environs. By virtue of their being first-generation health centers in the city administration, they are considered as role models for the new generation of health centers recently established in the city. Finding of this study can, therefore, be extrapolated to the new generation of health centers which are also providing the same level of service.

The average number of medicines per prescription was 2.0 which is within the range recommended by WHO [8]. One or more antibiotics were prescribed in 56.0% of the prescriptions reviewed with an average number of antibiotics per prescription of 0.61. This percentage of prescriptions containing antibiotics is far above the value recommended by WHO (not more than 30%) [8]. This figure is comparable with the national averages reported in the 2003 [9] and 2010 [10] national pharmaceutical sector assessments which reported percentage of antibiotics of 58% and 60%, respectively. However, the finding is much lower than the value (87.7%) reported for Addis Ababa City Administration in the 2003 national pharmaceutical sector assessment (87.7%) and the national value (62.3%) for health centers in the same study [9]. The present findings indicate the persistently high level of use of antibiotics in the city.

Comparing the finding with studies conducted in different parts of the country, the present finding is much lower than finding of the study (82.5%) conducted at health centers in Somali Regional State [11] but higher than the one (41.3%) reported by a study conducted at health centers in Bahir Dar City [13]. A study conducted at primary healthcare facilities in Wolkite Town [12] reported higher rate (63.0%) of antibiotics prescribing than the present finding. The present finding is comparable with findings reported from Nigeria [19] and Ghana [20] but higher than findings from studies conducted in African [21, 22] and Asian countries [23–26] which reported a value ranging from 21.1 to 51.5%.

Looking at the prescribing pattern, penicillins were the most frequently prescribed (51.9%) category of antibiotics followed by fluoroquinolones (18.3%) and sulphonamides (11.2%). Similar finding was reported from studies conducted at primary healthcare facilities in Turkey [27] and Malaysia [25] where penicillins accounted for 29.2% and 30.7% of the antibiotics prescribed, respectively. Cephalosporins and macrolide antibiotics were not frequently prescribed in the present study unlike the study conducted in Malaysia which reported cephalosporins (23.6%) and macrolides (16.0%) as the second and third commonly prescribed category of antibiotics [25]. In the present study, macrolides (azithromycin and erythromycin) were prescribed in only 1.4% of the cases.

By type of antibiotics, amoxicillin was the most frequently (44.8) prescribed antibiotic followed by ciprofloxacin (13.6%) and cotrimoxazole (11.2%). Though the percentage is high in the present study, similar findings were reported by studies conducted at primary healthcare facilities in Nigeria [19] and China [28] where amoxicillin was the most commonly prescribed antibiotic accounting for 25.4% and 21.3% of the prescribed antibiotics, respectively. Amoxicillin was also the most commonly prescribed antibiotic in studies conducted at health centers in Somali Regional State, Ethiopia [11], and at hospital in Southern Ethiopia [29] with amoxicillin accounting for 33.3% and 16.4% of the prescribed antibiotics.

Amoxicillin/clavulanic acid was the most commonly prescribed antibiotic (18.1%) in the study conducted in Turkey [27]. It is prescribed only in 2.7% of the cases in the present study. Only five antibiotics (amoxicillin, ciprofloxacin, cotrimoxazole, doxycycline, and norfloxacin) accounted for over 80% of the antibiotics prescribed in the current study indicating that few categories and types of antibiotics are being used repeatedly which can aggravate the emergence of AMR to these antibiotics.

Studies on antibacterial resistance have shown that emerging antibacterial resistance threatens the management of bacterial infections [6]. According to the study conducted in Southwest Ethiopia, the resistance rates of* S. aureus* and* S. saprophyticus* to ampicillin were 89.0% and 92.3%, respectively. The same group of bacterial isolates showed resistance to cotrimoxazole at rates of 82.3% and 89.0%, while for tetracycline the rates were 85.9% and 92.7%, respectively. Overall, multiple drug resistance was found to be 93.1%. A study from Eastern Ethiopia reported that isolates of* Salmonella* and* Shigella* were resistant to six commonly used antibiotics (ampicillin, amoxicillin, tetracycline, gentamicin, chloramphenicol, and norfloxacin) [5].

According to the antimicrobial sensitivity test conducted among pediatric patients at public health facilities in Addis Ababa, the overall resistance rates of isolated* Shigella *and* Salmonella *spp. were high for ampicillin (95.7% and 80.0%) and amoxicillin/clavulanic acid (91.4% and 80%), respectively. High sensitivity was observed among both isolates for ciprofloxacin (91.3%, 100%) and ceftriaxon (91.4%, 100%). More than 87% of* Shigella *and 70% of* Salmonella* species were resistant to two or more antibiotics (multiple resistance) [30]. Antimicrobial sensitivity study conducted among outpatients in Mekele Hospital (Northern Ethiopia) indicated that isolates of* Shigella *showed 100 and 66.7% resistance to ampicillin, amoxicillin, and cotrimoxazole respectively. Low levels of resistance were observed for norfloxacin and ciprofloxacin (6.7% each). Overall, 80% of the isolates showed multidrug resistance [31]. These findings call for cautious use of the existing antibiotics to ensure their continued use.

Most of the antibiotics (94.8%) were prescribed for oral administration which is encouraging in terms of patient safety and affordability. This finding is similar but higher in frequency as compared to the study conducted in a referral hospital in Northeast Ethiopia that reported oral route as the most common route accounting for 58% of the antibiotics prescribed [32]. Antibiotics were prescribed for parenteral administration in only 2.5% of the cases. The antibiotic prescribed for parenteral administration was ceftriaxone.

URTI was the most common diagnosis accounting for about a quarter (24.5%) of the diagnosis for which antibiotics were prescribed. URTI was also the first disease condition in the top ten morbidity list of all of the health centers surveyed accounting for 27.8 to 38.7% of the top 10 diseases according to the 2008 Ethiopian Calendar, EC (July 2015–June 2016) morbidity records of the health centers. This finding is comparable with studies done in Malaysia [25] and Yemen [33] which reported URTI as the most common clinical condition for antibiotics prescribing accounting for 49.2% and 38% of the cases, respectively. Almost 50% of the amoxicillin was prescribed for the treatment of URTIs.

Viruses play the most significant role in the pathogenesis of most URTIs. Typically, 70–80% of URTIs are triggered by viruses. However, 70–80% of patients with URTI worldwide are being prescribed antibiotics that are inconsistent with the causative organism. Thus, it is essential to differentiate between bacterial and viral infections to determine whether or not to give antibiotics [34]. The other common diagnoses like UTI, skin infections, and pneumonia were also among the top ten diseases in almost all of the health centers according to the 2008 EC morbidity records of the health centers. Antibiotics have contributed to significant proportion (46.0%) of the cost of medicines. Of this, URTI took over one-fifth of the cost of antibiotics prescribed. The cost of antibiotics was 50.2% of the total cost of medicines prescribed according to a study conducted in hospitals and primary healthcare facilities in Turkey [27]. Minimizing the prescribing of antibiotics for URTIs can, therefore, save significant amount of money in addition to preventing the emergence of AMR. Antibiotics like cotrimoxazole and ciprofloxacin were prescribed for unspecified type of diarrhea which might not require antibiotics.

The practice of ordering laboratory tests prior to prescribing antibiotics was very low and no AST was ordered to guide the prescribing of any of the antibiotics, even for UTIs. This indicates that most of the prescribing was done empirically without identifying the causative agent and looking at the antibiotics susceptibility patterns. This indiscriminate use of antibiotics can contribute a lot to the emergence of AMR. Addressing this issue through appropriate interventions by all concerned parties (health bureau, city administration health offices, the health facilities, and development partners) can contribute its part to the national effort in the prevention and containment of AMR.

Antibiotics prescribing was found to have significant association with patient's age where prescribing pattern decreased with increase in patient's age. This finding is comparable with findings of the study conducted in Yemen [33] which reported maximum percentage of antibiotics prescribing in pediatric patients and the least percentage of antibiotics was prescribed in patients over 60 years of age, but different from findings of the study conducted in Malaysia [25] where antibiotics were mostly prescribed for patients aged 20–39 years. Patient's sex and season of prescribing were not shown to have statistically significant association with antibiotics prescribing. In Malaysia, more female patients were given antibiotics in public clinics compared with male patients, while the reverse was true in private clinics [25].

The difference in the rate and patterns of antibiotic prescribing among different settings and geographic locations in the country might be due to difference in the prevalence of infectious diseases, differences in the qualifications and prescribing behaviors of prescribers, differences in availability of clinical guidelines, and the level of implementation of interventions targeted at promoting the rational use of antibiotics. Availability of diagnostic facilities and availability of antibiotics at the health facilities can also affect the rate and patterns of antibiotics prescribing.

Ethiopia has no separate guideline for the use of antibiotics. However, antibiotics are part of the standard treatment guidelines of the country used at different levels of care. There is a standard treatment guideline for health centers [35] which contains the guidelines for the use of antibiotics included in the list of medicines for health centers [18]. The guideline provides general guidance on common infectious diseases including definition, diagnosis, and nonpharmacological and pharmacological treatment options. The guideline does not provide adequate guidance on the specific uses of each of the antibiotics based on sensitivity data.

## 5. Limitations

As the study was conducted in few of the public health centers having long experience in service provision, the findings cannot be generalized to all health centers found in the city. Since the study was conducted retrospectively, data incompleteness due to inaccessibility of prescriptions and medical charts or because of prescriptions that are not dispensed or documented in the health centers might have introduced some bias in the study. Data on laboratory investigations were taken from medical charts only by looking at the date of prescribing of the antibiotics and hence laboratory investigation that might have been done before or after the date of prescribing might have been missed. Since there is limited data on antibiotics prescribing at primary healthcare facilities both locally and globally, some of the findings were compared with findings of studies conducted at hospitals or mix of different levels of health facilities.

## 6. Conclusion

There was high rate of antibiotics prescribing and antibiotics have contributed for significant proportion of the cost of medicines prescribed with wide variation among the health centers. Significant proportion of antibiotics are being prescribed for minor conditions like URTI which are expected to be of viral origin most of the time. This might exacerbate the AMR situation in the country and cost. Targeted interventions should be designed and implemented to improve the prescribing of antibiotics in the health centers. Large-scale study should be conducted to fully understand the rate and patterns of antibiotics prescribing in the city and identify the associated factors and thereby comprehensively address the problem.

## Figures and Tables

**Table 1:** Patient-related information of prescriptions dispensed at health centers in Addis Ababa, January 1–December 31, 2016.

Variable	Frequency	Percent
Patient's sex (*n* = 898)		
Male	350	39.0
Female	548	61.0
Patient's age in years (*n* = 888)		
0–14	222	25.0
15–34	370	41.7
35–54	168	18.9
≥55	128	14.4
Prescribing quarter (*n* = 900)		
1st quarter (Jan.–March, 2016)	223	24.8
2nd quarter (April–June, 2016)	219	24.3
3rd quarter (July–Sept., 2016)	230	25.6
4th quarter (Oct.–Dec., 2016)	228	25.3

**Table 2:** The rate of antibiotics prescribing by patient characteristics and season of prescribing at health centers in Addis Ababa, January 1–December 31, 2016.

Variable	Frequency	Percent
Patient's sex		
Male	194	55.4
Female	310	56.6
Patient's age in years		
0–14	155	69.8
15–34	218	58.9
35–54	79	47.0
≥55	46	35.9
Prescribing quarter		
1st quarter (Jan.–March, 2016)	124	55.6
2nd quarter (April–June, 2016)	111	50.7
3rd quarter (July–Sept., 2016)	137	59.6
4th quarter (Oct.–Dec., 2016)	132	57.9

**Table 3:** Frequently prescribed type and category of antibiotics at health centers in Addis Ababa, January 1–December 31, 2016.

Variable	Frequency	Percent	Cumulative percent
*Category of antibiotics *(*n* = 553)			
Penicillins	287	51.9	51.9
Fluoroquinolones	101	18.3	70.2
Sulphonamides	62	11.2	81.4
Tetracyclines	55	9.9	91.3
Metronidazole	17	3.1	94.4
Cephalosporins	14	2.5	96.9
Macrolides	8	1.4	98.3
Chloramphenicol	8	1.4	99.7
Aminoglycosides	1	0.2	100.0
*Type of antibiotics *(*n* = 553)			
Amoxicillin	248	44.8	44.8
Ciprofloxacin	75	13.6	58.4
Cotrimoxazole	62	11.2	69.6
Doxycycline	48	8.7	78.3
Norfloxacin	26	4.7	83.0
Cloxacillin	24	4.3	87.3
Metronidazole	17	3.1	90.4
Amoxicillin + clavulanic acid	15	2.7	93.1
Ceftriaxone	13	2.4	95.5
Chloramphenicol	8	1.4	96.9
Azithromycin	7	1.3	98.2
Tetracycline	7	1.3	99.5
Cephalexin	1	0.2	99.6
Erythromycin	1	0.2	99.8
Gentamycin	1	0.2	100.0

**Table 4:** Common diagnosis for frequently prescribed antibiotics (as a single medicine) at health centers in Addis Ababa, January 1–December 31, 2016.

Antibiotics and diagnosis	Frequency	Percent
Amoxicillin		
URTI	106	49.5
Tonsillitis	39	18.2
Topical infections	15	7.0
Trauma	12	5.6
UTI	10	4.7
Amoxicillin + clavulanic acid		
Pneumonia	4	28.6
Tonsillitis	2	14.3
URTI	2	14.3
Ciprofloxacin		
Typhoid fever	13	25.5
Gastroenteritis	13	25.5
AFI	8	15.7
Diarrhea	5	9.8
Doxycycline		
AFI	13	50.0
Typhoid fever	8	30.8
Bronchitis	2	7.7
Cotrimoxazole		
Diarrhea	15	29.4
Gastroenteritis	12	23.5
UTI	12	23.5
Topical infections	5	9.8
Norfloxacin		
UTI	21	100

**Table 5:** Bivariate logistic regression of factors associated with antibiotics prescribing at health centers in Addis Ababa, January 1–December 31, 2016.

Variable	Antibiotics prescribed?		COR (95% CI)
	No	Yes	
Patient's sex			
Male	156	194	0.955 (0.729–1.251)
Female	238	310	1.000
Age of patient (years)			
0–14	67	155	**4.124 (2.601**–**6.539)**
15–34	152	218	**2.557 (1.686**–**3.876)**
35–54	89	79	1.582 (0.988–2.535)
≥55	82	46	1.000
Prescribing quarter			
1st quarter (Jan.–March, 2016)	99	124	1.000
2nd quarter (April–June, 2016)	108	111	0.821 (0.564–1.193)
3rd quarter (July–Sept., 2016)	93	137	1.176 (0.810–1.708)
4th quarter (Oct.–Dec., 2016)	96	132	1.098 (0.756–1.594)
